# A neutralizable dimeric anti-thrombin aptamer with potent anticoagulant activity in mice

**DOI:** 10.1016/j.omtn.2023.07.038

**Published:** 2023-08-02

**Authors:** Masanobu Nagano, Kazuki Kubota, Asuka Sakata, Rei Nakamura, Toru Yoshitomi, Koji Wakui, Keitaro Yoshimoto

**Affiliations:** 1 Department of Life Sciences, Graduate School of Arts and Sciences, The University of Tokyo, 3-8-1 Komaba, Meguro, Tokyo 153-8902, Japan; 2 Medicinal Biology of Thrombosis and Hemostasis, Nara Medical University, 840 Shijo-cho, Kashihara, Nara 634-8521, Japan

**Keywords:** MT: Oligonucleotides: Therapies and Applications, aptamer, thrombin, avidity, antidote, heparin-induced thrombocytopenia, bivalent aptamer, circular aptamer, TBA29, iv injection

## Abstract

Heparin-induced thrombocytopenia (HIT) is a complication caused by administration of the anticoagulant heparin. Although the number of patients with HIT has drastically increased because of coronavirus disease 2019 (COVID-19), the currently used thrombin inhibitors for HIT therapy do not have antidotes to arrest the severe bleeding that occurs as a side effect; therefore, establishment of safer treatments for HIT patients is imperative. Here, we devised a potent thrombin inhibitor based on bivalent aptamers with a higher safety profile via combination with the antidote. Using an anti-thrombin DNA aptamer M08s-1 as a promising anticoagulant, its homodimer and heterodimer with TBA29 linked by a conformationally flexible linker or a rigid duplex linker were designed. The dimerized M08s-1-based aptamers had about 100-fold increased binding affinity to human and mouse thrombin compared with the monomer counterparts. Administration of these bivalent aptamers into mice revealed that the anticoagulant activity of the dimers significantly surpassed that of an approved drug for HIT treatment, argatroban. Moreover, adding protamine sulfate as an antidote against the most potent bivalent aptamer completely suppressed the anticoagulant activity of the dimer. Emerging potent and neutralizable anticoagulant aptamers will be promising candidates for HIT treatment with a higher safety profile.

## Graphical abstract

**Figure:**
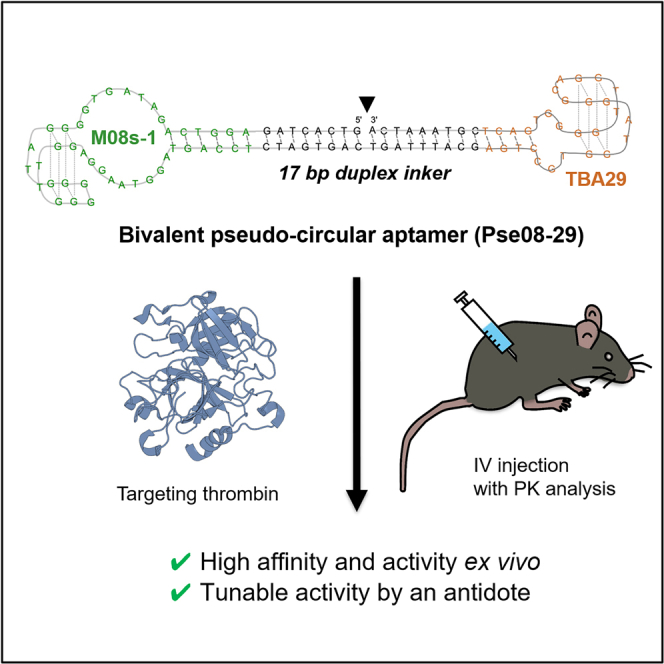

## Teaser

Yoshimoto and colleagues report a highly potent anticoagulant aptamer in mice. A bivalent aptamer dimerized with M08s-1 showed potent anticoagulant activity in mice, and an antidote could reverse the activity. This aptamer may be an alternative to current drugs for patients with heparin-induced thrombocytopenia drastically increased by COVID-19.

## Introduction

Heparin is a naturally occurring heparan sulfate and the first anticoagulant agent in history.^1^^,^^2^ Because heparin is in widespread clinical use not only for treatment of serious thromboembolisms, such as heart attack and disseminated intravascular coagulation, but also for prevention of thrombosis as a result of kidney dialysis, extracorporeal membrane oxygenation (ECMO), or cardiopulmonary bypass machines, it is included in the World Health Organization (WHO) Model List of Essential Medicines as an effective medicine needed in a healthcare system.^3^

Any heparin therapy can induce heparin-induced thrombocytopenia (HIT) as a severe complication in up to 0.2%–3% of patients.^4^^,^^5^^,^^6^^,^^7^ HIT is caused by the immune response to a complex of heparin with platelet factor 4 (PF4), a neoantigen, which then drives abnormal production of activated coagulant factor II, referred to as thrombin. The hypercoagulable state induced by thrombin results in life-threatening arterial or venous thrombosis with development of stroke, myocardial infarction, and deep vein thrombosis (DVT). It is recommended that patients severely affected by coronavirus disease 2019 (COVID-19) receive heparin for prophylaxis of thrombosis in an ECMO machine or for treatment of thrombosis incurred as a complication, implying that the potential number of patients with HIT is increasing globally.^8^^,^^9^^,^^10^

HIT treatment is performed via an intravenous drip infusion of thrombin inhibitors, such as a small molecule and a peptide, argatroban and bivalirudin (Figure S1).^5^^,^^11^ However, when severe life-threatening bleeding occurs during HIT treatment with conventional thrombin inhibitors, the only way to reverse the anticoagulant effect is to cease the infusion; persistent bleeding for longer than the half-lives (around 30 min to 1 h) of the inhibitors is a considerable burden for patients.^10^ Therefore, developing an anticoagulant that can immediately neutralize the effects when necessary is critical for achieving safe treatment of HIT.

**Figure 1:**
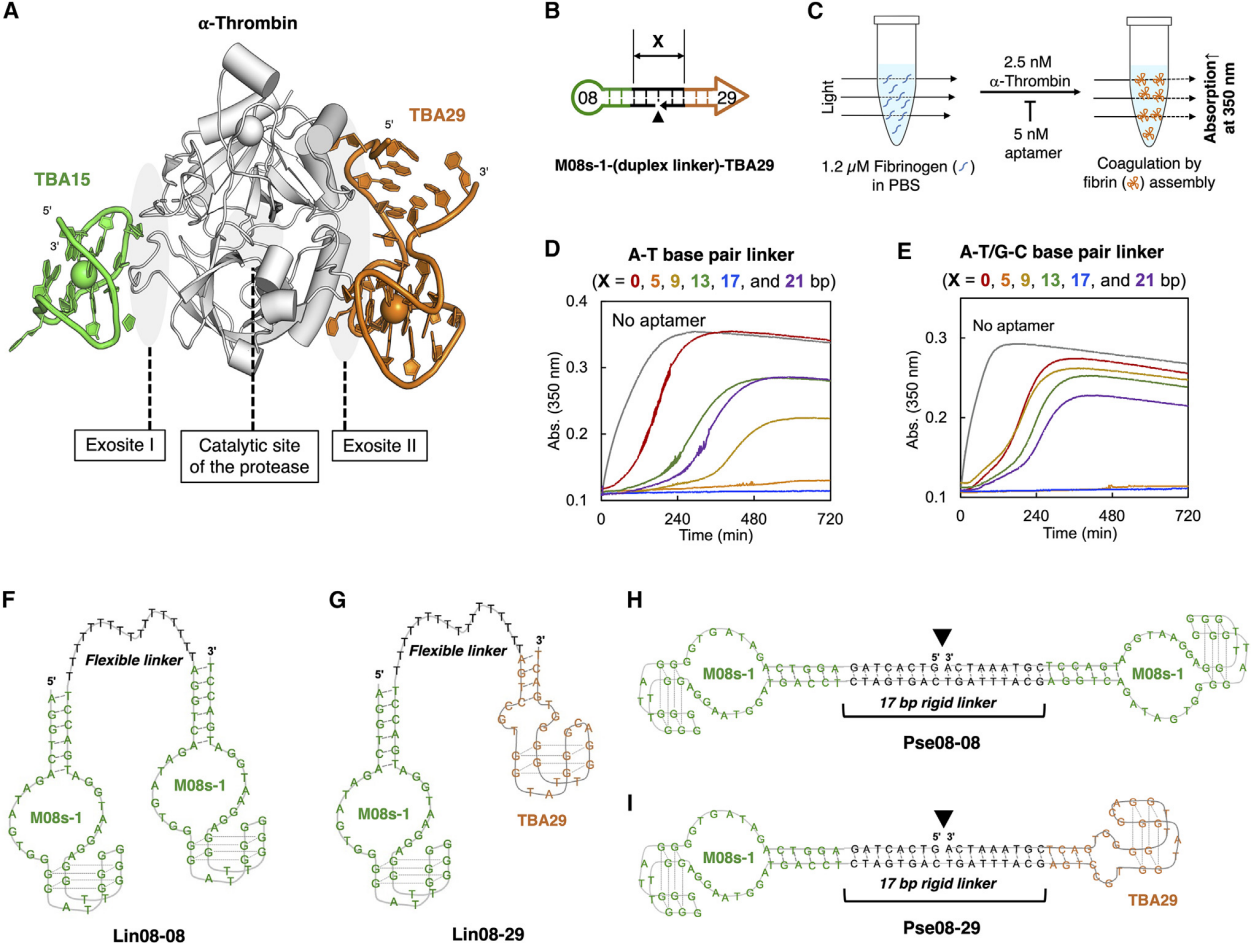
Optimization of duplex linker length with the M08s-1 variant and TBA29 (A) The ternary complex of human thrombin with TBA15 and TBA29 (PDB: 5EW1). (B) Schematic representation of the duplex linker (X is the number of base pairs). (C) Clotting time assay to see the thrombin inhibition of the aptamer. (D) Length dependency of the duplex linker composed of A-T base pairs. (E) Length dependency of the duplex linker composed of A-T/G-C mixed base pairs. (F–I) Secondary structures of M08s-1 containing bivalent anti-thrombin aptamers in this study: Lin08-08, Lin08-29, Pse08-08, and Pse08-29. The secondary structure of M08s-1 is an estimated structure based on previous studies and the Quadruplex forming G-Rich Sequences (QGRS) mapper.^23^

Nucleic acid aptamers are ligands composed of a single-stranded oligonucleotide (single-stranded DNA [ssDNA] or RNA) possessing a high binding affinity and specificity to a target of interest.^12^^,^^13^ Because the complement sequence against such an aptamer is a highly specific, low-cost, and rapidly eliminated antidote, development of a neutralizable anticoagulant, a combination of the bioactive aptamer and its antidote, has long been a research target.^13^^,^^14^^,^^15^^,^^16^ TBA15 (HD1) is the first reported anti-thrombin DNA aptamer, and many anti-thrombin aptamers have been reported since (Figure S2A).^17^^,^^18^^,^^19^ TBA15 exerts its anticoagulant activity by preventing fibrin formation via binding to exosite I of thrombin and blocking fibrinogen binding (Figure 1A).^20^^,^^21^ Currently, the only anti-thrombin aptamer in a clinical trial is NU172, an updated version of TBA15, investigated for treating off-pump coronary artery grafting bypass, with no reports for other diseases (Figure S2C).^22^ One of the reasons for this narrow disease scope is that the *in vivo* anticoagulant activity of NU172 and different, relatively new anti-thrombin aptamers has not been fully investigated. Therefore, ones with higher anticoagulant activity *in vivo* than NU172 will be suitable candidates for HIT therapy and treatment for other diseases.

Recently, we discovered an anti-thrombin DNA aptamer, M08s-1, using systematic evolution of ligands by exponential enrichment (SELEX) with microbead-assisted capillary electrophoresis (MACE) and revealed that M08s-1 possesses higher anticoagulant activity than NU172 (Figure S2E).^24^^,^^25^^,^^26^ Despite the promising anticoagulant activity *in vitro*, the anticoagulant activity in an animal model has not been assessed. Furthermore, M08s-1 has sufficient structural scope to enhance anticoagulant activity via dimerization.^27^^,^^28^ For these reasons, we are motivated to investigate the *in vivo* anticoagulant activity and reversal of the activity of M08s-1 and its dimers to develop a neutralizable drug candidate for HIT.

Here, we designed four M08s-1-based bivalent aptamers, where the linker between the monomeric aptamer was constructed with classic flexible poly-deoxythymidine (dT) or a rigid duplex. Both linker types of bivalent aptamers showed approximately 100-fold higher affinity to human and mouse thrombin than the monomeric counterpart M08s-1. Intravenous injection of these aptamers into mice showed significantly stronger anticoagulant activity than argatroban and NU172. Moreover, the anticoagulant activity of the discovered bivalent dimers could be partially but strongly reversed by a short complementary strand and even readily neutralized by protamine sulfate, indicating that they are potential alternatives to the drugs currently used for HIT therapy.

## Results

### Design of M08s-1-based bivalent anti-thrombin aptamers

To increase the potency of the thrombin inhibitor aptamer M08s-1 *in vivo*, harnessing avidity by dimerization is a simple and robust way to achieve it. So far, a heterodimeric aptamer, HD1-22, in which TBA29 (HD22) as an anti-thrombin aptamer targeting another exosite (exosite II) of thrombin^29^^,^^30^ is linked to TBA15 via a conformationally flexible single-stranded linker, has been well studied (Figure 1A).^31^ Later, the optimal single-stranded linker between TBA15 and TBA29 was optimized by *in vitro* selection, resulting in that single-strand linker folding into a conformationally rigid duplex by taking a pseudo-circular structure.^32^ Because M08s-1 binds to exosite I of thrombin,^33^ similar to TBA15, we designed a heterodimer of M08s-1 and TBA29 with the duplex linker and optimized the length and composition of the linker (Figures 1B and S3A). Screening of the duplex linker was performed based on thrombin clotting time (TCT) with purified fibrinogen, where fibrin cleaved from fibrinogen by thrombin spontaneously aggregates to increase the turbidity of the solution as a result of “clotting”; inhibition of thrombin by the aptamer prolongs the clotting time (Figure 1C).^34^ When the duplex linker was composed of base pairs with homo-dT and homo-dA chains, where the base pair length was variable (X = 0, 5, 9, 13, 17, and 21 bp in Figure 1C), the clotting was nearly completely inhibited by a linker length with 5 and 17 bp, reflecting high anticoagulant activity via thrombin inhibition (Figure 1D). Next, after changing the composition of the duplex linker from homo-dT homo-dA chains to the mixed base pairs of A:T and G:C based on a previously reported duplex linker,^35^ a linker length of 5 and 17 bp was also critical for anticoagulant activity, among others (Figure 1E). These results suggest that inhibition of thrombin by the heterodimer of M08s-1 and TBA29 with a duplex linker is independent of the linker sequence but strictly dependent on the specific linker length. Because the 17-bp length showed more potent inhibition activity than the 5-bp linker, we selected the 17-bp linker. Furthermore, the linker compositions were determined to be mixed A:T and G:C base pairs because of high thermodynamic stability compared with the homo-dA:homo-dT duplex. Therefore, we designed M08s-ds_17_-TBA29 (Pse08-29), having our optimized duplex linker as a heterodimer of M08s-1 and TBA29, aiming for a potent pseudo-circular bivalent aptamer *in vivo* (Figure 1I).

Because the inhibition activity is not derived from TBA29 but M08s-1, examination of a homodimer of M08s-1 is also needed in addition to pse08-29 to investigate the best dimer with high anticoagulant activity. Furthermore, *in vivo,* the structural properties of the linker-connecting aptamers would also affect behavior; therefore, a classic flexible linker should also be investigated. Finally, we designed three M08s-1-based bivalent aptamers with M08s-dT_17_-M08s and M08s-dT_17_-TBA29^36^ (Lin08-08 and Lin08-29) with the classic linear of 17 mer dT linker (Figures 1F and 1G), and M08s-ds_17_-M08s (Pse08-08) with a rigid linker with 17-bp duplex for further experiments (Figure 1H).

### Affinity evaluation of dimeric anti-thrombin aptamers

Next, we evaluated the affinity of the bivalent aptamers to human and mouse thrombin using surface plasmon resonance (SPR) using accociation rate constant (*k*_a_), dissociation rate constant (*k*_d_), and dissociation constant (K_D_). Upon immobilization of thrombin onto a carboxymethylated matrix, the response unit (RU) was set to a low value (500) to bias the 1:1 binding of the dimeric aptamer to thrombin and avoid multiple binding modes (Figures S4C–S4D). All dimeric aptamers were readily prepared from the corresponding ssDNAs using an annealing procedure. With human thrombin, although M08s and TBA29 bound with a similar dissociation constant (K_D_) of 47.2 and 36.9 nM, respectively (Table 1; Figure S5A), whereas M08s-1 homodimers, Lin08-08, and Pse08-08 increased their affinity to almost 100-fold that of the monomer counterpart independent of the linker type (K_D_ = 0.4 nM). Pse08-29, the heterodimer with a rigid linker, showed 15-fold higher affinity than the monomer counterparts but weaker affinity than M08s-1 homodimers, presumably because of the distant binding site between exosite I and II (Figures S4C and S4D).

**Table 1:** Evaluation of affinity of anti-thrombin aptamers to thrombin by SPR

Aptamer	Human thrombin			Mouse thrombin		
	*k*_a_ (1/Ms)	*k*_d_ (1/s)	K_D_ (nM)	*k*_a_ (1/Ms)	*k*_d_ (1/s)	K_D_ (nM)
M08s	7.04 × 10^5^	3.33 × 10^−2^	47.2	3.56 × 10^5^	1.76 × 10^−1^	495
TBA29	1.34 × 10^5^	4.94 × 10^−3^	36.9	2.76 × 10^5^	6.07 × 10^−3^	22.0
Lin(08-08)	1.63 × 10^6^	6.94 × 10^−4^	0.4	6.41 × 10^5^	4.30 × 10^−3^	6.7
Pse(08-08)	1.19 × 10^6^	5.10 × 10^−4^	0.4	7.35 × 10^5^	3.06 × 10^−3^	4.2
Pse(08–29)	1.33 × 10^6^	3.47 × 10^−3^	2.6	6.72 × 10^5^	4.26 × 10^−3^	6.3

Human or mouse thrombin was immobilized to the CM5 chip with a low RU of 500. The K_D_ of Pse(08–29) was calculated as 1:1 binding by approximating that M08s and TBA29 possess the same K_D_ as the homodimer.

Understanding the binding affinity of aptamers to mouse thrombin is important for performing mouse dosing experiments. The binding affinity of monomeric M08s-1 to mouse thrombin had a 10-fold decreased K_D_ value compared with that of human thrombin (47.2 vs. 495 nM) (Table 1; Figure S5B). However, TBA29 showed similar affinity regardless of the species difference (36.9 nM vs. 22 nM). Although the affinity of the M08s-1 homodimers decreased 10-fold compared with that against human thrombin, this still maintained a 100-fold higher binding affinity to the monomer counterparts (K_D_ = 495 nM vs. 6.7 and 4.2 nM, respectively), indicating that the considerable anticoagulant activity of dimeric aptamers would be observed in mice. The pseudo-circular heterodimer Pse08-29 maintained a similarly high binding affinity to human thrombin (K_D_ = 2.6 nM vs. 6.3 nM). Although determination of the K_D_ of Lin08-29 with the flexible poly(dT) linker was not feasible because of the complex binding modes (Figure S4E), an avidity analysis based on the k_off_ value was performed to compare all dimers in parallel, which determined that the degree of avidity of Lin08-29 was similar to that of other dimers (Figure S6).^37^ Collectively, the dimeric aptamers possessed high binding affinity to one of the monomer counterparts. The loss of the binding affinity to mouse thrombin for M08s-1 could be overcome by increased binding affinity via dimerization, suggesting the value of testing the anticoagulant activity in mice.

### Evaluation of the anticoagulant activity of M08s-1-based bivalent aptamers by activated partial thromboplastin time (aPTT) in spiked plasma

**Figure 2:**
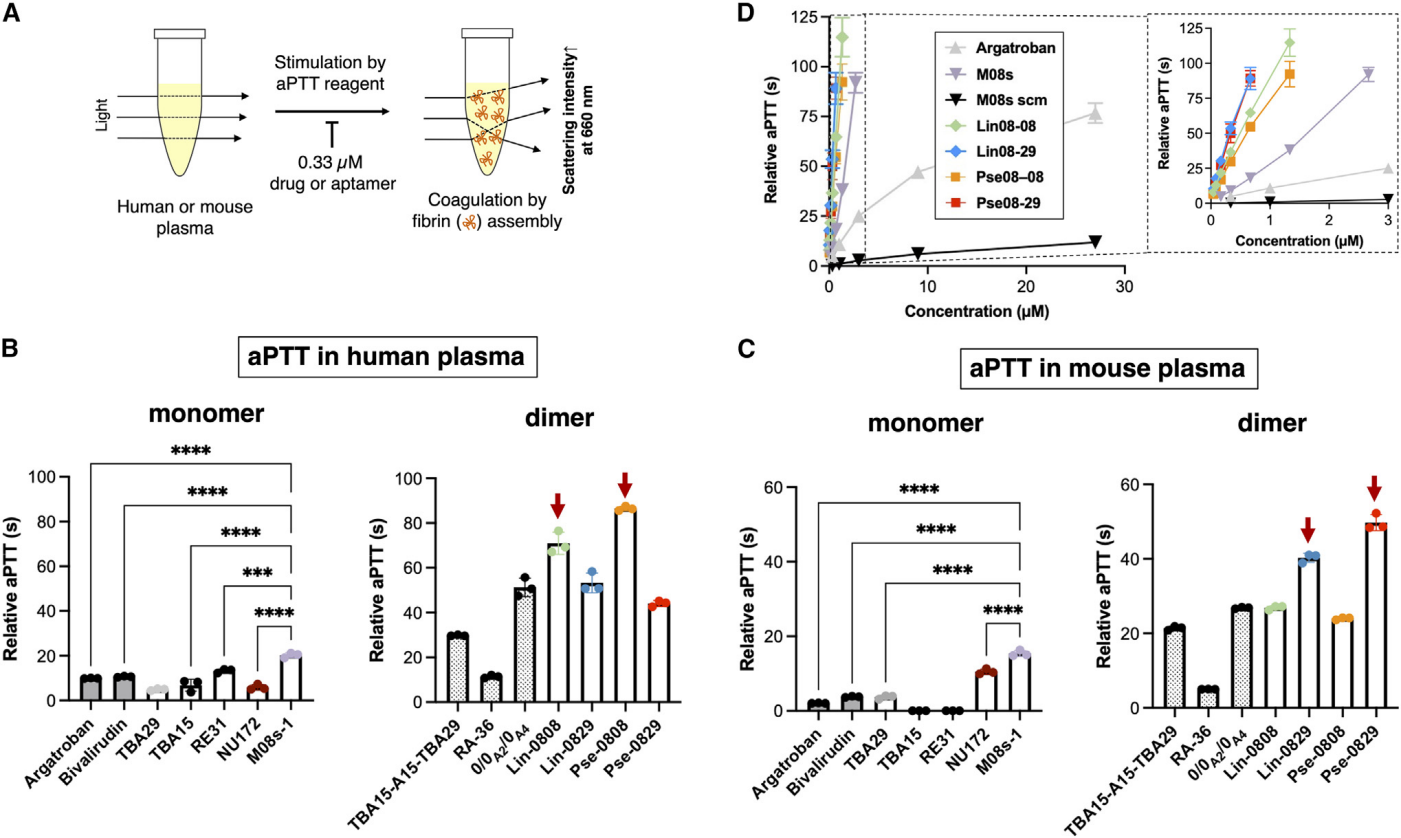
Analysis of anticoagulant activity of aptamers and drugs using aPTT in spiked plasma (A) Schematic of aPTT. (B) The aPTT in human plasma. (C) The aPTT in mouse plasma. (D) Concentration dependency of aptamers on the anticoagulant activity in mouse plasma. The clotting time of the aptamer (−) as a control in human plasma and mouse plasma was 27.1 and 25.1 s, and the relative clotting time at the y axis was calculated by subtraction of the averaged value of the control gained in triplicate. Error bars indicate SD (N = 3). The statistical significance was tested using a t test. ∗∗∗p < 0.001, ∗∗∗∗p < 0.0001.

With the confirmation of high binding affinity of dimeric aptamers to thrombin, we next assessed the anticoagulant activity in plasma by measuring aPTT, a clinically used monitoring method for parenteral anticoagulant drugs such as heparin and argatroban.^38^^,^^39^ Notably, the aPTT assay differs from the simple TCT with purified fibrinogen (Figure 1C) because the aPTT assay allows assessment of the outcome of the coagulation cascade from all coagulant factors in an intrinsic pathway (Figures 2A and S7). For instance, exosite I is also exposed on prothrombin, where binding of the aptamers to exosite I can reduce the rate of prothrombin activation and could prolong aPTT without having a direct effect on thrombin activity in plasma;^40^^,^^41^ therefore, aPTT reflects inhibition of thrombin and prothrombin. The “relative aPTT” was calculated by subtracting the aPTT of PBS spiked in human plasma (27 s) from the aPTT of a tested sample spiked in human plasma. We first investigated the anticoagulant activity of the monomeric M08s-1 and compared this with the known anti-thrombin DNA aptamers TBA13, TBA29, RE31,^42^ and NU172 (Figures S2A–S2D); in addition, the argatroban and bivalirudin anti-thrombin drugs indicative of HIT were tested (Figures S1A and S1B). Measurement of the drugs and monomeric aptamers at molecular equivalence (0.33 μM) in human plasma using the aPTT assay demonstrated a relative aPTT of M08s-1 at 20 s, which was the longest of those tested and was greater than that of previously reported aptamers and even of drugs (Figure 2B). This tendency was also observed in mouse plasma (Figure 2C), suggesting that M08s-1 at a monomer level was the most potent thrombin inhibitor among the tested aptamers and drugs.

We next compared the M08s-1-based dimers with the known dimeric aptamers RA-36,^43^ TBA15-dA_15_-TBA29,^44^ and 0/0_A2_/0_A4_,^45^ which are a homodimers of TBA15 linked with a single dT linker, a classic heterodimer with TBA15 and TBA29 attached by a flexible poly(dA) linker, and a rationally designed heterodimer with RE31 and TBA29 with a rigid duplex linker, respectively (Figures S2F–S2H). The aPTT assay in human plasma showed that the two heterodimers, Lin08-29 and Pse08-29, exhibited a similar degree of prolongation of the relative aPTT than the previously reported heterodimer 0/0_A2_/0_A4_ (relative aPTT = 49 s). However, the two M08s-1-homodimers, Lin08-08 and Pse08-08, showed 70 s and 85 s of relative aPTT, respectively (Figure 2B). When the therapeutic window of unfractionated heparin, which is in the 2- to 3-fold range against naive aPTT as a control (27 s),^46^ is used for evaluation of the aptamers, the therapeutic window in relative aPTT can be defined to be a range of 27–54 s, suggesting that both M08s-1-homodimers showing aPTTs of ∼85 s bear enough effectiveness. For mouse plasma, compared with the aPTT of dimers, the M08s-1-based-heterodimers Lin08-29 and Pse08-29 showed a higher anticoagulant effect (40 s and 50 s of relative aPTT) than M08s-1-homodimers and other previously reported dimers (Figure 2C).

We next evaluated the concentration dependency of the aptamers and argatroban for the anticoagulant activity in mouse plasma (Figure 2D). Although argatroban required approximately 12 μM to reach the maximum therapeutic window of 50 s of relative aPTT, M08s-1 could reach the time at a 3-fold lower concentration (4 μM). Furthermore, four M08s-1-based bivalent aptamers nearly reached 50 s of relative aPTT at 0.3–0.45 μM, suggesting that these aptamers were effective at 30-fold and 10-fold lower concentrations compared with argatroban and monomeric M08s-1, respectively. Because M08s-1-scm, whose nucleobase sequence were designed by scrambling that of M08s-1, did not reach the maximum therapeutic window of more than 30 μM, the working range of the dimeric aptamers at a low 0.3–0.45 μM was likely the outcome of structured M08s-1 unit to thrombin and prothrombin in plasma. Overall, the homodimers Lin08-08 and Pse08-08 and heterodimers Lin08-29 and Pse08-29 showed the strongest anticoagulant activity in human plasma or mouse plasma, among others, suggesting that dimerization based on M08s-1 was a reasonable approach to produce potent anticoagulant activity.

### Assessment of anticoagulant activity of the bivalent aptamers *in vivo* using aPTT

**Figure 3:**
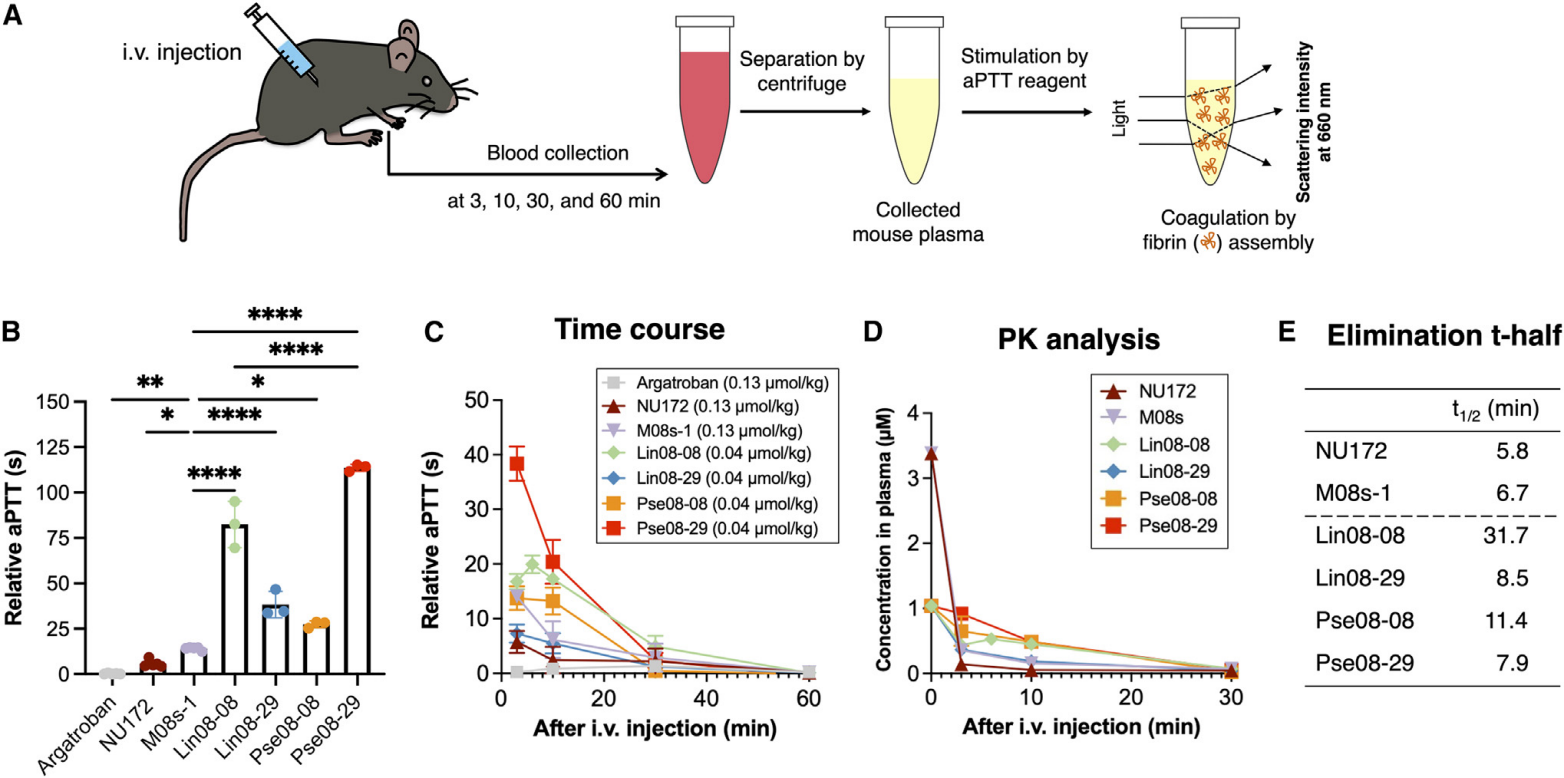
Anticoagulant activity of the aptamers after their systemic administration to mice (A) Schematic of the experiment. (B) The maximum anticoagulant effect at the 3-min time point using a uniform dose of 1.3 μmol/kg. The statistical significance was tested using a t test. ∗p < 0.1, ∗∗p < 0.01, ∗∗∗∗p < 0.0001. (C) Time course of clotting time plotted against collected serum after the injection at several time points. Relative clotting time was obtained by subtracting the treated data by 22.6 s, an averaged value of aptamer (−) as control aPTT in a triplicate. (D) Pharmacokinetics (PK) study of the administered aptamers, where the concentration in mouse plasma was calculated based on the standard curve from anticoagulant activity in spiked mouse plasma (Figure 2D). Argatroban was not calculated because of lack of aPTT time. (E) The half-lives (t-half) of the aptamers are based on PK analysis.

Because the M08s-1-based dimeric aptamers showed high anticoagulant activity using the aPTT assay with spiked plasma, we investigated their activity in mice. This experiment followed the way of clinical monitoring, where plasma from collected blood samples after injection of the aptamers was assessed using the aPTT assay (Figure 3A). Although argatroban and bivalirudin are administered by continuous infusion in the clinic, in this study, the aptamers were injected at a single dose level to investigate the efficacy and pharmacokinetics in advance. To do this, argatroban, NU172, M08s-1, and four M08s-1-bivalent aptamers (Lin08-08, Lin08-29, Pse08-08, and Pse08-29) were administered separately to mice via intravenous bolus injection at molar equivalents (0.13 μmol/kg dose). Then, the aPTT 3 min after injection was defined as the maximum aPTT, where all bivalent aptamers showed a more prolonged relative aPTT than argatroban or the monomeric aptamers NU172 and M08s-1 (Figure 3B). The heterodimer with a rigid linker, Pse08-29, showed the highest anticoagulant activity among M08s-1-based dimers. Next, mice were dosed with monomeric aptamers and argatroban at 0.13 μmol/kg dose and four M08s-1-dimeric aptamers at 0.04 μmol/kg, and blood was collected until 1 h after administration. Despite their lower injection doses than the monomers and argatroban, all dimeric aptamers showed high anticoagulant activity based on the aPTT (Figure 3C).

Besides aPTT, prothrombin time (PT) is also measured for approved direct thrombin inhibitors (DTIs), including argatroban.^47^ Therefore, we performed the PT assay in the same manner via mouse injections (Figures S7, S8A, and S8B). Among the aptamers, M08s-based Lin08-08 and Pse08-29 prolonged the PT slightly longer than others, which is the same trend as with aPTT. In all cases, the effects of aPPT were significant compared with PT, which was the same trend as for DTIs seen with the therapeutic dose.^48^

Conversion of relative aPTT to molar concentration based on the standard curves shown in Figure 2D can estimate the aptamer pharmacodynamics (Figure 3D). Two monomeric aptamers, NU172 and M08s-1, were rapidly diminished in the distribution phase after 3 min and behaved in a biphasic manner. In contrast, the dimers acted more in a monophasic manner. The β-phase elimination half-lives in blood were less than 10 min, although the homodimers, Lin08-08 and Pse08-08, showed slightly longer half-lives up to 31 min (Figure 3E). Collectively, intravenous administration of the aptamers and argatroban to mice followed by aPTT testing revealed that the heterodimer pse08-29 with the rigid duplex linker had the most potent anticoagulant activity in mice.

### Serum stability of bivalent anti-thrombin aptamers

Chemically unmodified aptamers are known to be labile against nucleases in blood. To better understand the mechanism of fast clearance *in vivo*, the stability of the monomeric M08s-1 and four dimeric aptamers (Lin08-08, Lin08-29, Pse08-08, and Pse08-29) in 50% human or mouse serum was investigated (Figures S10 and S11). The half-lives of all the aptamers in human and mouse serum were greater than 30 min, implying that aptamer degradation *in vivo* was not the main reason for the rapid activity loss, likely because of distribution to tissues or clearance to the kidneys.^49^^,^^50^

### Neutralization of the anticoagulant activity of the bivalent aptamers

**Figure 4:**
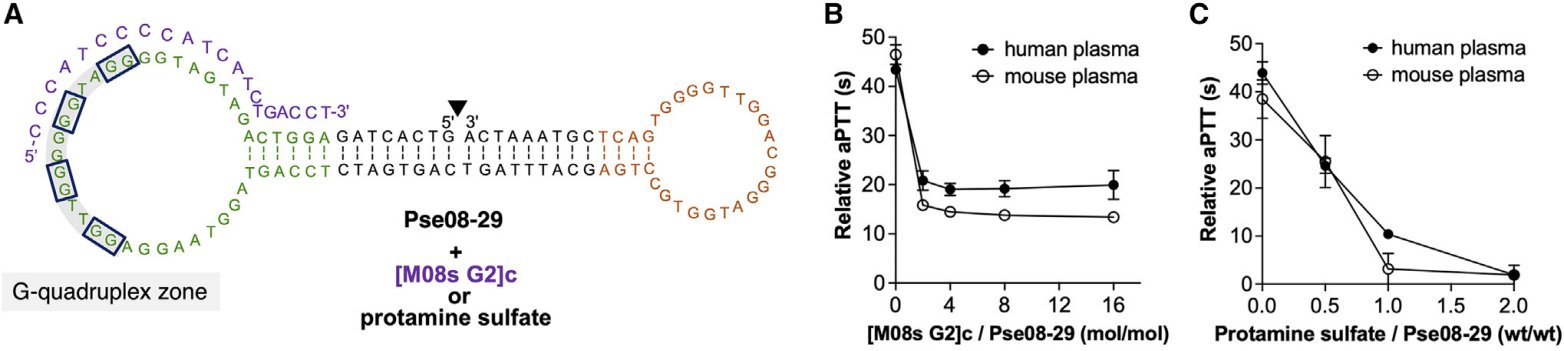
Neutralization of the anticoagulant effect by antidotes (A) Secondary structure of Pse08-29 and its partial complement sequence, [M08s G2]c, as an antidote. The solid black line inside gray is deoxyguanines predicted G-quadruplex structures suggested by QGRS mapper as G-sore, 36. (B) Neutralization assay with a short complementary strand assessed by aPTT. Pse08-29; 0.33 μM, complementary strand; 0, 0.66, 1.32, 2.64, or 5.28 μM. (C) Neutralization assay with protamine sulfate assessed by aPTT. Pse08-29; 5.6 mg/mL, protamine; 2.8, 5.6, or 11.2 mg/mL. Error bars indicate SD (N = 3).

Even if aptamers have a short half-life, developing an antidote to rapidly reverse their anticoagulant effects in severe bleeding in HIT patients is crucial. To achieve our goal of developing a neutralizable thrombin-inhibitory aptamer, we screened optimal antidote sequences from 9 short complementary strands against the M08s-1 site of Pse08-29, which showed the most potent anticoagulant activity in mice (Figures S12A and S12B). Mixing of antidotes with a 16-fold concentration of 0.33 μM of Pse08-29 in human serum followed by aPTT, the assessment identified [M08s-1 G2]c (22-mer, entry 3) as having the most efficient reversal of relative aPTT from 43 s to 19 s. Then the dose dependency of [M08s-1 G2]c was tested in aPTT and revealed that it plateaued at a 4-fold concentration in the aPTT assay (Figures 4A and 4B). This suggested that the complementary sequence of the M08s-1 site on Pse08-29 could not completely reverse the anticoagulation effect of the aptamer.

It is reported that some anticoagulant aptamers can be neutralized with protamine sulfate, the antidote to heparin.^51^^,^^52^ Therefore, we wanted to investigate whether polycationic protamine sulfate could be used as a reversing agent for the bivalent aptamer Pse08-29 bearing a negatively charged phosphate backbone. Protamine sulfate successfully reversed the anticoagulant effect of Pse08-29 in a dose-dependent manner. It almost completely reversed at an aptamer: antidote ratio of 1:2 (w/w), restoring the aPTT to baseline (Figure 4C). Thus, not only the complementary strand but also protamine sulfate were found to be valuable antidotes to control the activity of Pse08-29, which is vital for mitigating the bleeding risks associated with argatroban and bivalirudin used in HIT therapy.

## Discussion

Developing a thrombin inhibitor with high anticoagulant activity and its antidote is an important issue for HIT therapy. The recent COVID-19 pandemic raised the number of patients with HIT because of the increased use of heparin; unfortunately, currently used drugs for HIT have no antidotes to arrest bleeding as a side effect. In this study, we devised M08s-1-based bivalent aptamers with higher anticoagulant activity than other aptamers and even current drugs used for HIT. We demonstrated that an antidote could reverse the activity and is useful for developing safer drugs for HIT treatment.

Although many dimeric anti-thrombin aptamers have been reported, most are heterodimers, where TBA15 targeting exosite I and TBA29^53^ targeting exosite II of thrombin are linked with conformationally flexible poly(dA) or poly(dT) linkers as a hinge for bivalent tight binding (Figures 1A and S2B).^31^^,^^44^^,^^54^^,^^55^ Ahmad et al.^32^ used *in vitro* selection to optimize the linker composition between TBA15 and TBA29, resulting in a conformationally rigid duplex-type linker in which the overall structure becomes a pseudo-circular (dumbbell-like) bivalent aptamer with a nick. Based on this knowledge, screening of the length of the duplex linker between M08s-1 and TBA29 resulted in high anticoagulant activity with a linker with 5 or 17 bp. This trend is presumably due to the 12-bp difference in length between them. Because a helical pitch of the DNA duplex is approximately 10 bp,^56^ these 5- and 17-bp lengths may match the topology of the two aptamers to induce tight binding to thrombin by forming a 2:2 or 3:3 complex other than standard 1:1 or 2:1 formation (Figure S3B).^45^ Finally, we designed four M08s-1-based bivalent aptamers with a 17-bp rigid duplex (Pse08-08 and Pse08-29) or flexible poly(dT) linker (Lin08-08 and Lin08-29) to identify the optimal linker for M08s-1 dimers.

In the experimental results obtained from SPR analysis, there was a difference in species specificity for the binding affinity, in which the one of monomeric M08s-1 to human thrombin reduced 10-fold compared with the mouse thrombin. The reason why the affinity of M08s-1 differed between human and mouse thrombin may be because the exosite I of human thrombin targeted by M08s-1 could be structurally different from that of mouse thrombin. This hypothesis was supported by alignment of the amino acid sequences of both thrombins, where the mouse thrombin sequence contained many differences in the exosite I region (Figure S9), and a similar result between human and mouse thrombin was also seen in the inhibition assay for TBA15 targeting exosite I.^57^ Overall, the bivalent aptamers yielded a 100-fold higher affinity than the monomeric counterpart to human and mouse thrombins, which benefit from retaining the activity *in vivo*.

The *in vitro* aPTT assay in spiked plasma revealed that monomeric M08s-1 had higher anticoagulant activity than currently used drugs for HIT treatment and other identified aptamers. The anticoagulant activity of the designed dimers was approximately 10-fold higher than that of M08s-1. The performance of the M08s-1-homodimers in human plasma using the aPTT assay was superior to that of the M08s-1-based-heterodimers. Intriguingly, the result in mouse plasma was the opposite, likely because of the influence of decreased affinity of M08s-1 from human to mouse thrombin identified in the SPR experiment (Table 1).

The aPTT assay with the plasma of mice after administration of the bivalent aptamers suggested that the anticoagulant activity significantly surpassed that of the approved drugs for HIT and the clinical candidate aptamers argatroban and NU172, respectively. Two monomeric aptamers, NU172 and M08s-1, were rapidly diminished in the distribution phase after 3 min and behaved in a biphasic manner, whereas the dimers behaved in a monophasic manner; this difference was probably due to the effect of the different molecular weights (8–13 kDa vs. 35–38 kDa) on their filtration by the kidneys (Figure 3D).^58^ This residence trend of the dimeric aptamers within 3 min after administration would also be the reason for having high anticoagulant activity in mice compared with the monomeric aptamers. The β-elimination half-life of the aptamers was less than 10 min, except for M08s-1-based homodimers, which were similar to that of the reported unmodified aptamer (Figure 3E).^50^ A long half-life in mice was seen in M08s-homodimeric aptamers, especially Lin08-08, up to 31 min; however, the ones of the other dimers and monomers remained up to 8.5 min, which is typical behavior for aptamers (Figure 3E). Although a chemically modified therapeutic aptamer, pegaptanib, which targets vascular endothelial growth factor (VEGF) for ocular vascular disease, prolonged the aPTT as a side effect because of the nonspecific interaction with plasma proteins at a 5 mg/kg dose,^59^^,^^60^ the M08s-1-based bivalent aptamers were chemically unmodified and showed sufficient efficacy at the lower dose of 0.04 mg/kg, suggesting less risk for nonspecific prolongation of aPTT. Although the reasons for the potent anticoagulant activity *in vivo* of pse08-29 compared with the other three bivalent dimers are unknown, our study suggests that the keys to the high potency of the discovered bivalent aptamer, Pse08-29, would be at least (1) the selection of M08s-1, (2) the avidity harnessed by dimerization, (3) the high binding affinity of TBA29 to mouse thrombin, and (4) the high residence in blood at the distribution phase.

Argatroban and bivalirudin, used to treat HIT, have no antidote, increasing the risk of the treatment. Therefore, designing an antidote to Pse08-29, which showed high anticoagulant activity *in vivo,* is crucial. We designed nine complementary sequences of the M08s-1 site on Pse08-29, but they could not completely reverse the anticoagulation effect. Likely, Pse08-29 linked by a rigid duplex linker may benefit the high thermodynamic stability and high anticoagulant activity, however, it could lose structural flexibility to be hijacked by complementary strands. In turn, using clinically approved protamine sulfate as an antidote for heparin successfully suppressed the high anticoagulant activity of pse08-29, strengthening the utility of the bivalent aptamer with existing drugs for HIT without an antidote. Further *in vivo* evaluations with bleeding and prevention of thrombus formation are currently underway.

## Materials and methods

All oligonucleotides at high performance liquid chromatography (HPLC) grade were purchased from Eurofins Genomics (Japan) (Table S1). Before use, each 150 μM aptamer in PBS was annealed by heating at 95°C for 3 min using a dry-bath incubator (Major Science, Taiwan) and cooling immediately in a heat block previously stored at 25°C. The annealed aptamer was diluted in PBS for the desired concentrations. All solutions were prepared using ultrapure water from a Milli-Q water purification system (Merck Millipore, USA). Human fibrinogen, mouse serum, 2-amino-2-hydroxymethyl-1,3-propanediol; Tris, EDTA-2Na-2H_2_O, 40 w/v% acrylamide/bis mixed solution 19:1, ammonium persulfate (APS), *N,N,N,N′*-tetramethylethylene; TEMED, 10w/v% polyoxyethylene(20) sorbitan monolaurate solution; 10% Tween 20, Dulbecco’s PBS (−), urea, and protamine sulfate from salmon were purchased from FUJIFILM Wako Pure Chemicals Industries (Japan). Normal human serum and normal mouse plasma were purchased from Cosmo Bio (Japan). Human thrombin and bivalirudin trifluoroacetate salt were purchased from Sigma-Aldrich (MO, USA). Mouse thrombin (Creative Biomart, NY, USA), argatroban monohydrate (TCI, Japan), normal human plasma (George King Bio-Medical, KS, USA), and 10 mM sodium acetate (pH 5.5; Cytiva, MA, USA) were used as received. C57BL/6 mice were purchased from Japan SLC (Shizuoka, Japan).

### Inhibitory activity analysis of the aptamers in thrombin and fibrinogen mixed solution

The experiment was performed based on a previous report.^36^ Briefly, to a 0.6-mL tube, 100 μL of pre-annealed 12.5 nM aptamer in PBS and 100 μL of 6.25 nM human thrombin in PBS were added and incubated at room temperature for 15 min. 80 μL of the mixture was added to each well of a 96-well Costar® assay plate (Corning, WA, USA). Immediately after adding 20 μL of 6 μM human fibrinogen in PBS to each well of assay plate using an 8-serial pipettor, the clotting curve was measured as an increase in absorbance at 350 nm associated with fibrin gel formation using a UV-visible microplate reader (Epoch, Agilent Technologies, CA, USA). The initiation point of the coagulation was determined by Origin 7.0 software. The experiments were performed in duplicate.

### Affinity analysis of anti-thrombin aptamers using SPR

The running buffer for SPR analysis (PBS [pH 7.4], 0.05 [v/v%] surfactant Tween 20) was filtered using Sartolab® RF 1000 (Sartorius, Germany). Pre-annealed aptamers in SPR running buffer were filtered through an Acrodisc® syringe filter (Paul, WA, USA). SPR spectroscopy-based binding analysis was performed using a Biacore X100 (Cytiva, MA, USA). 1 μM human thrombin and mouse thrombin in 10 mM sodium acetate (pH 5.5) were coupled on a CM5 sensor chip CM5 (Cytiva, UK) by 1-ethyl-3-(3-dimethylaminopropyl)carbodiimide hydrochloride (EDC)/*N*-hydroxysuccinimide (NHS) chemistry at a flow rate of 10 μL min^−1^ in SPR running buffer until the RU reached 500. Using single-cycle kinetics, the concentration series of the aptamers was injected using 1 M NaCl solution as the regeneration buffer. The obtained data were fitted with a 1:1 binding model using the Biacore X100 evaluation software (Cytiva, UK), and the dissociation constant (K_D_) was calculated.

### aPTT assay

#### aPTT in spiked plasma

1.6 μL of each pre-annealed 30 μM aptamers in PBS and 48.4 μL of normal human plasma or normal mouse plasma were incubated at 37°C for 1 min using a fully automated blood coagulation instrument (CA-620; Sysmex, Japan). Then, 50 μL of aPTT reagent (Thrombocheck; Sysmex) was added and further incubated at 37°C for 2 min. Then, 50 μL of 0.025 M calcium chloride was added, and aPTT was measured immediately by tracking the change in scattered light intensity over time at 660 nm. The experiments were performed in triplicate. The statical analysis was performed using Tukey’s test and ordinary one-way ANOVA (GraphPad Prism v.9.4.1).

#### aPTT in plasma from aptamer-injected mice

50 μL of the collected plasma sample stored at −147°C was thawed and incubated at 37°C for 1 min using a fully automated blood coagulation instrument (CA-620, Sysmex). Then, 50 μL of aPTT reagent (Thrombocheck, Sysmex) was added and further incubated at 37°C for 2 min. Then, 50 μL of 0.025 M calcium chloride was added, and aPTT was measured immediately by tracking the change in scattered light intensity over time at 660 nm.

### Injection into mice and collection of plasma samples

C57BL/6 mice (22 g, 7 weeks old, n = 3 or 5) were anesthetized by inhalation of isoflurane (Viatris, Canonsburg, PA, USA) and injected with pre-annealed aptamer in PBS with 0.04 μmol/kg (16 μM, 50 μL) or 0.13 μmol/kg (52 μM, 50 μL) by bolus intravenous injection via jugular vein, and blood was collected from the inferior vena cava 3, 10, 30, or 60 min after administration. Collected blood samples were immediately mixed with 3.8% sodium citrate (Muto Pure Chemicals, Tokyo, Japan) (blood:3.8% sodium citrate = 9:1 [v/v]) and centrifuged at 1,500 × *g* for 15 min at 25°C using a centrifuge (Thermo Fisher Scientific, MA, USA), and then the plasma layer was collected and stored at −147°C. Mice were euthanized by cervical dislocation under isoflurane anesthesia after collection of blood at each time point.

### PT assay in plasma from aptamer-injected mice

50 μL of the collected plasma sample stored at −147°C was thawed and incubated at 37°C for 1 min using a fully automated blood coagulation instrument (CA-620, Sysmex). Then, 100 μL of PT reagent (Thrombocheck, Sysmex) was added and further incubated at 37°C for 2 min. Then, 50 μL of 0.025 M calcium chloride was added, and PT was measured by tracking the change in scattered light intensity over time at 660 nm.

### Pharmacodynamics analysis

The aPTT resulting from the administered aptamers at each time point were converted into concentrations based on standard curves generated from the *in vitro* anticoagulant effect from spiked mouse plasma in Figure 2D. The concentration at time points of 3, (6), 10, 30, and 60 min were subjected to one-phase decay using GraphPad Prism v.9.4.1. to analyze their *in vivo* half-lives.

### Serum stability assessment of anti-thrombin aptamers

The experiment was performed based on a previous study.^61^ In a 0.6-mL low-absorption tube, 40 μL of pre-annealed 4 μM aptamer in PBS was mixed with 40 μL of normal human serum or 40 μL of normal mouse serum and then incubated at 37°C using a cool incubator (Ikuta Industry, Japan). The sample solution was quenched by adding 20 μL of 100 mM EDTA-2Na-2H_2_O solution and refrigerated at 4°C until its analysis. Then, 100 μL of 2× loading buffer containing 8 M urea, 2 mM EDTA-2Na-2H_2_O, and 2 mM Tris was added and heated at 95°C for 5 min using a dry-bath incubator (Major Science). After denaturation, the samples were subjected to electrophoresis using 12% acrylamide gel containing urea at 200 V for 30 min. Then, the gel was stained by Gel Star Nucleic Acid Gel Stain (Lonza), and the band derived from the aptamer was visualized by UV irradiation using Fusion Solo S (Vilber Lourmat, France). The bands were quantified by Fusion Solo 6S Edge software (Vilber Lourmat).

### Neutralization analysis of the anticoagulant activity using aPTT in plasma

48.3 μL of normal human plasma (George King Bio-Medical), 0.83 μL of a 60 μM pre-annealed aptamer (final concentration: 0.333 μM, 5.6 μg/mL), and 0.83 μL of 120, 240, 480, or 960 μM complement sequence was incubated at 37°C for 1 min using a fully automated blood coagulation instrument (CA-620, Sysmex). Then, 50 μL of aPTT reagent (Actin FSL, Sysmex) was added and further incubated at 37°C for 2 min. Then, 50 μL of 0.025 M calcium chloride was added, and aPTT was measured immediately by tracking the change in scattered light intensity over time at 660 nm. In the case of protamine sulfate as an antidote, 0.83 μL of 2.8, 5.6, or 11.2 μg/mL protamine sulfate in PBS was used instead of the complement sequence. The experiments were performed in triplicate.

**Ethical information**

All experiments using mice were conducted in accordance with the institutional guidelines approved by the Nara Medical University Institutional Animal Care and Use Committee (13239 and 13339).

## Data and code availability

All data generated during this study are included in this published article and its supplemental information or available upon request.

## Supplemental information

Document S1. Figures S1–S12 and Table S1

Document S2. Article plus supplemental information
