# Supplementary material for: A neutralizable dimeric anti-thrombin aptamer with potent anticoagulant activity in mice
Source: Mol Ther Nucleic Acids. 2023 Aug 2;33:762–72. doi: 10.1016/j.omtn.2023.07.038 (PMC10445101; doi:10.1016/j.omtn.2023.07.038)
Supplement: Document S1. Figures S1–S12 and Table S1 [file mmc1.pdf]

OMTN, Volume *33*

Supplemental information

A neutralizable dimeric anti-thrombin aptamer with potent anticoagulant activity in mice

Masanobu Nagano, Kazuki Kubota, Asuka Sakata, Rei Nakamura, Toru Yoshitomi, Koji Wakui, and Keitaro Yoshimoto

a

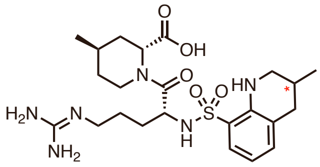

Argatroban

Molecular Weight: 508.64

b

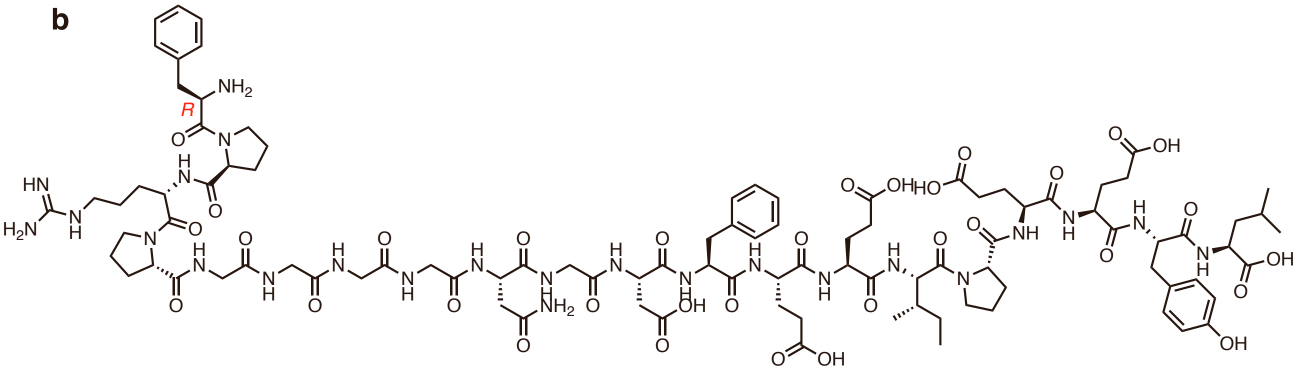

D-Phe-Pro-Arg-Pro-Gly-Gly-Gly-Gly- Asn-Gly-Asp-Phe-Glu-Glu-Ile- Pro-Glu-Glu-Tyr-Leu

Bivariludin

Molecular Weight: 2180.32

Figure S1. Structures of currently used drugs for HIT treatment. (a) Argatroban, which bind to a catalytic site of thrombin to inhibit the activity. FDA approved argatroban in 2002. (b) Bivariludin, which binds both the catalytic site and exosite-I of α-thrombin and show strong anticoagulant activity. Bivariludin was approved for HIT in 2019 by FDA.

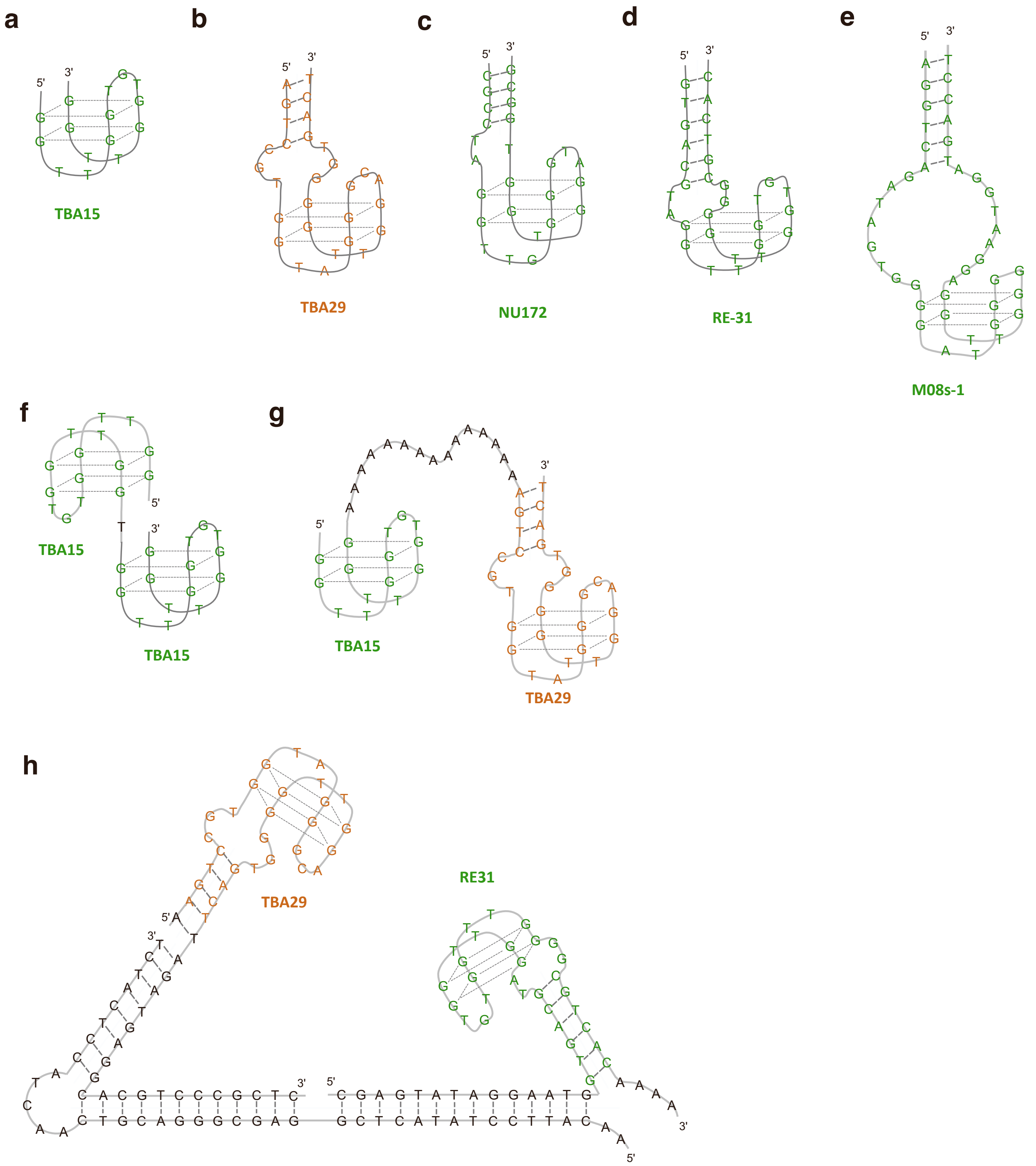

Figure S2. Secondary structures of DNA aptamers used in this study. (a) TBA15, (b) TBA29, (c) NU172, (d) RE31, (e) M08s-1, (f) RA-36, (g) TBA15-A15-TBA29, and (h) 0/0$_{A2}$/0$_{A4}$. Secondary structure of M08s-1 is estimated structure based on previous study and RGRS mapper and the others were derived from co-crystal structures bound to human α-thrombin.

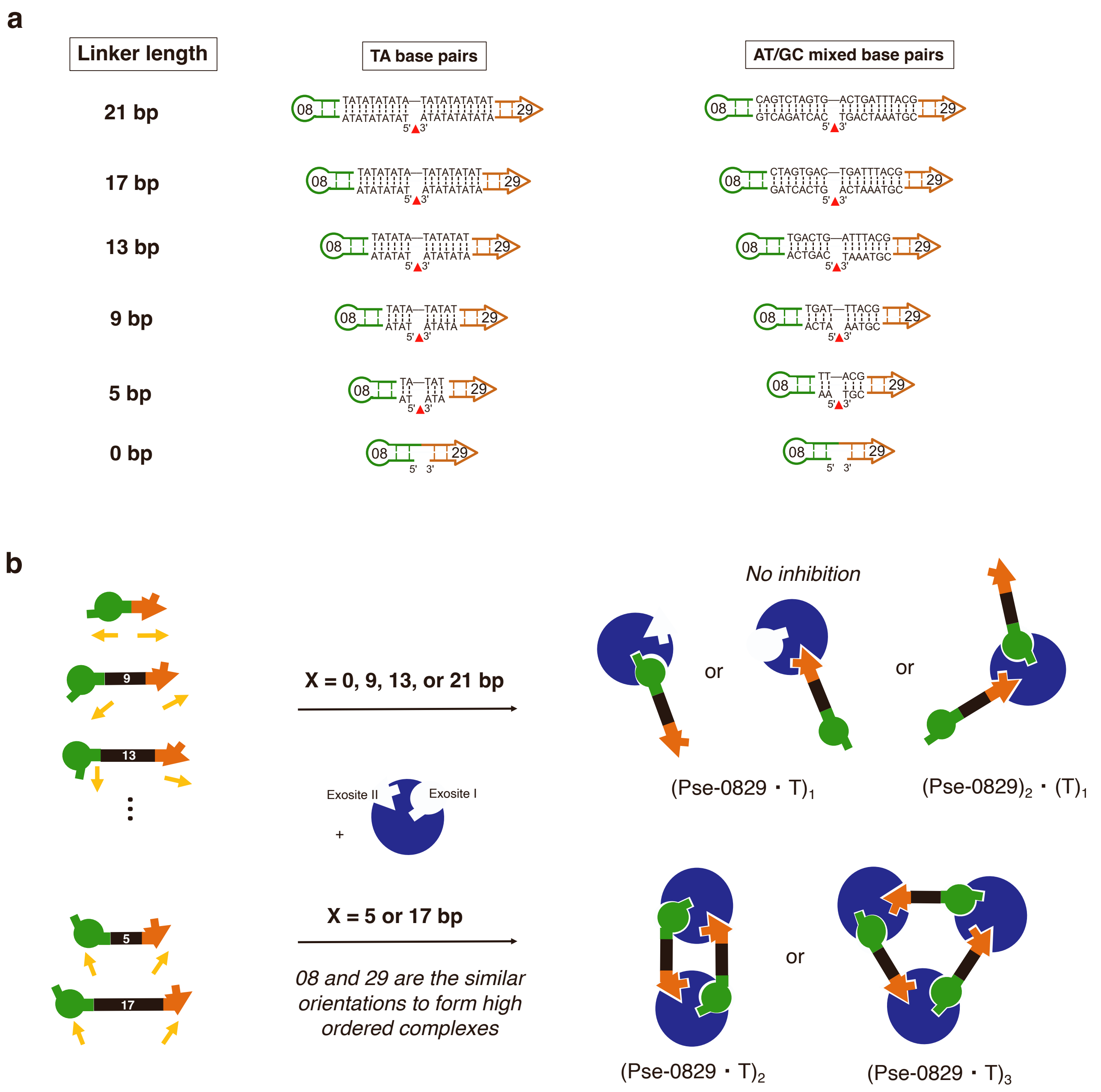

Figure S3. Optimization of duplex's linker length. (a) Secondary structures of dumbbell-shaped dimer with different sequences and length. (b) Possible mechanism for strong inhibition activity of the Pse08-29 with 5 bp and 17 bp linkers. These would be either 2:2 or 3:3 complex forms and when an inappropriate, rigid linker doesn't allow two aptamer moieties to simultaneously bind to the same a-Thrombin. AT/GC duplex linker was selected based on previous report.[1]

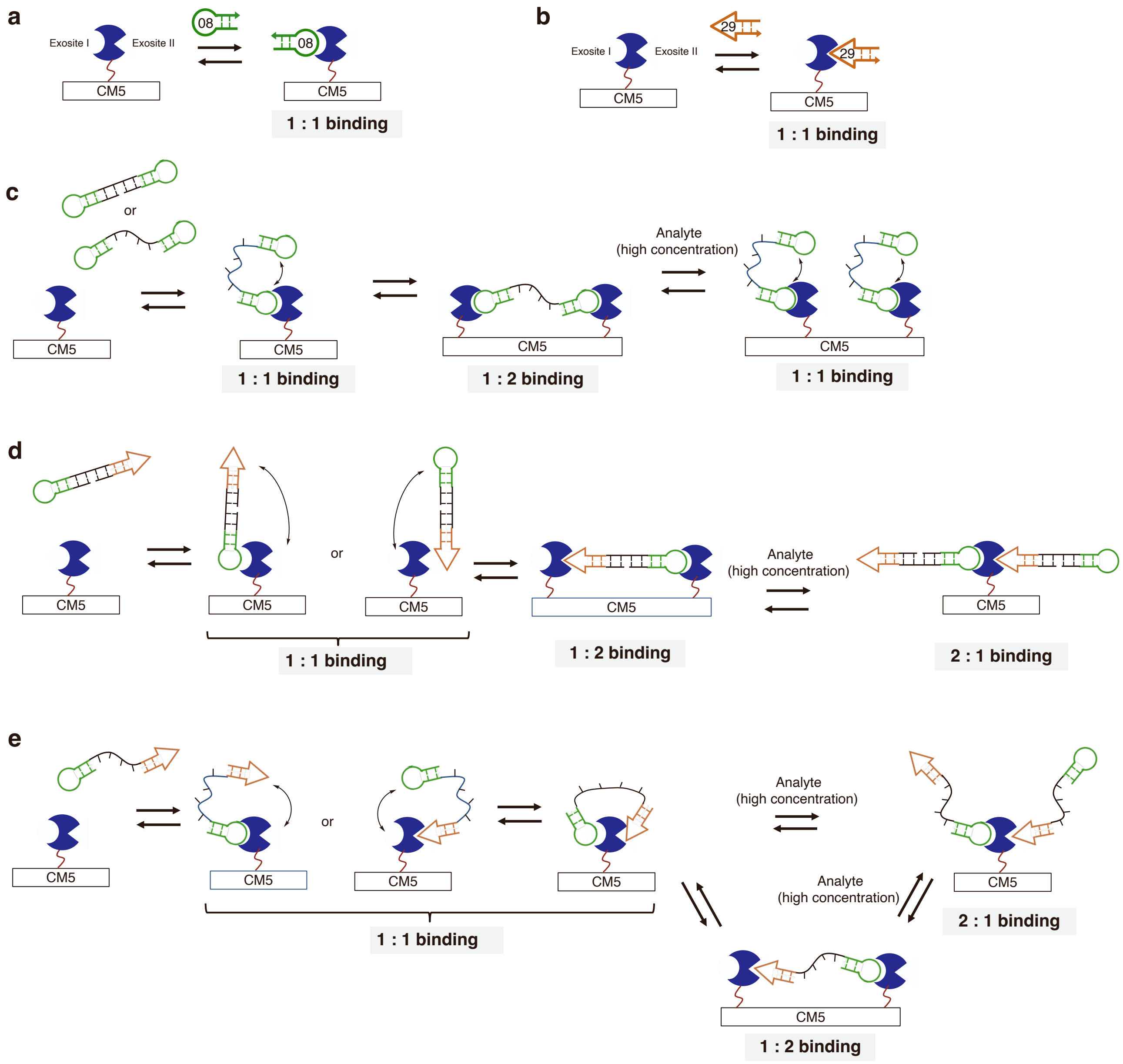

Figure S4. Possible binding modes of dimeric aptamers in SPR. (a) A binding mode of M08s-1 to exosite-I of α-thrombin. (b) A binding mode of TBA29 to exosite-II of α-thrombin. (c) Binding modes of M08s-1's homodimers to the exosite-I of α-thrombin. (d) Binding modes of rigid biparatopic aptamer Pse08-29 to α-thrombin. (e) Binding modes of Lin08-29 as a biparatopic aptamer with flexible poly-dT linker to a-thrombin.

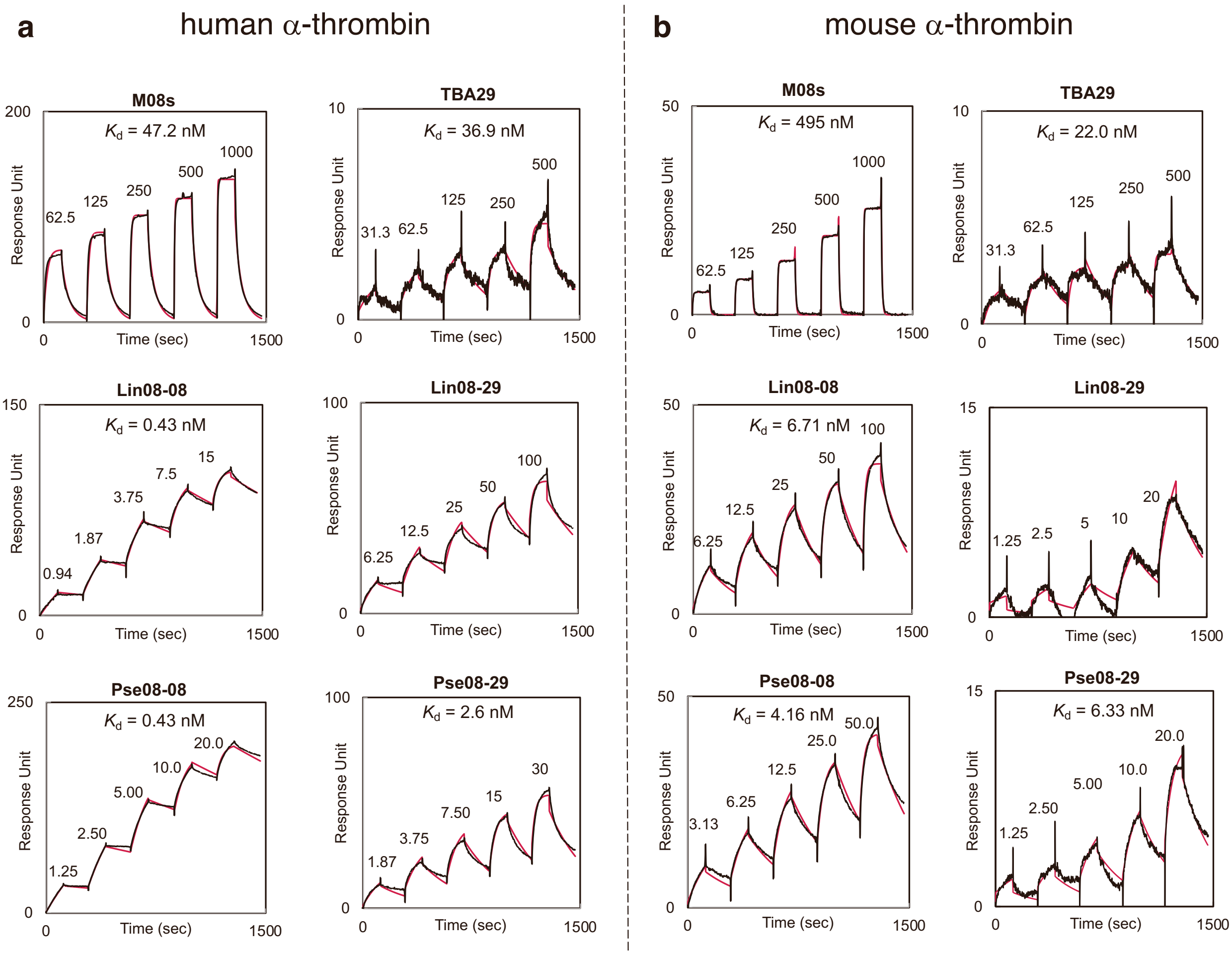

Figure S5. Affinity analysis of the aptamers by SPR. (a) Kinetics titration against human α-thrombin (b) Kinetics titration to mouse α-thrombin. Red, fitting curve; black, experimental curve. In all the experiments, a-thrombin was immobilized on CM5 chip as a ligand.

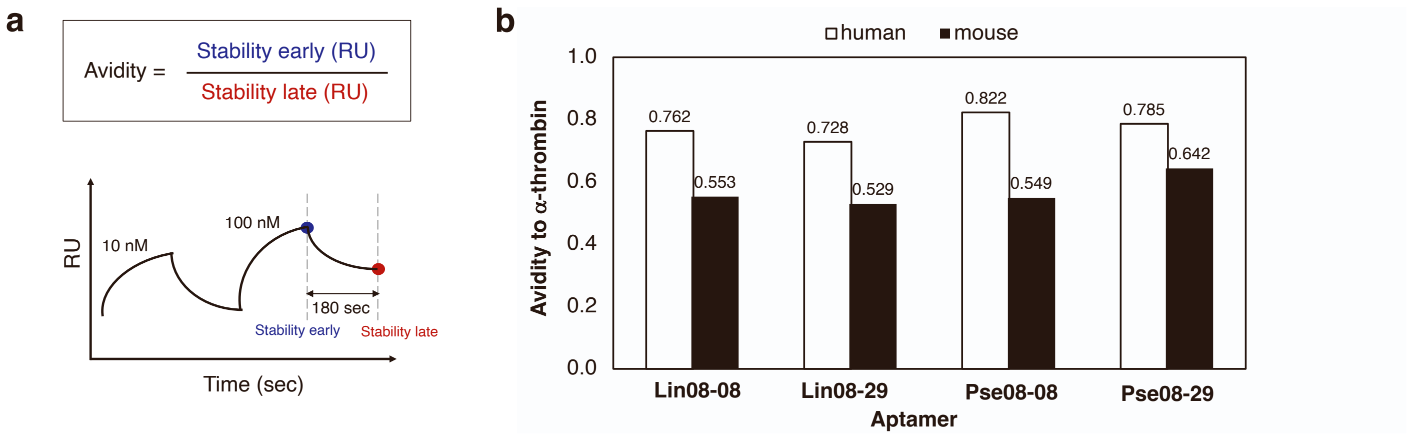

Figure S6. Avidity evaluation of dimeric aptamers to a-thrombin based on SPR. (a) Calculation of acidity using the final dissociation step in single kinetics. (b) Comparison of avidity between dimeric aptamers.

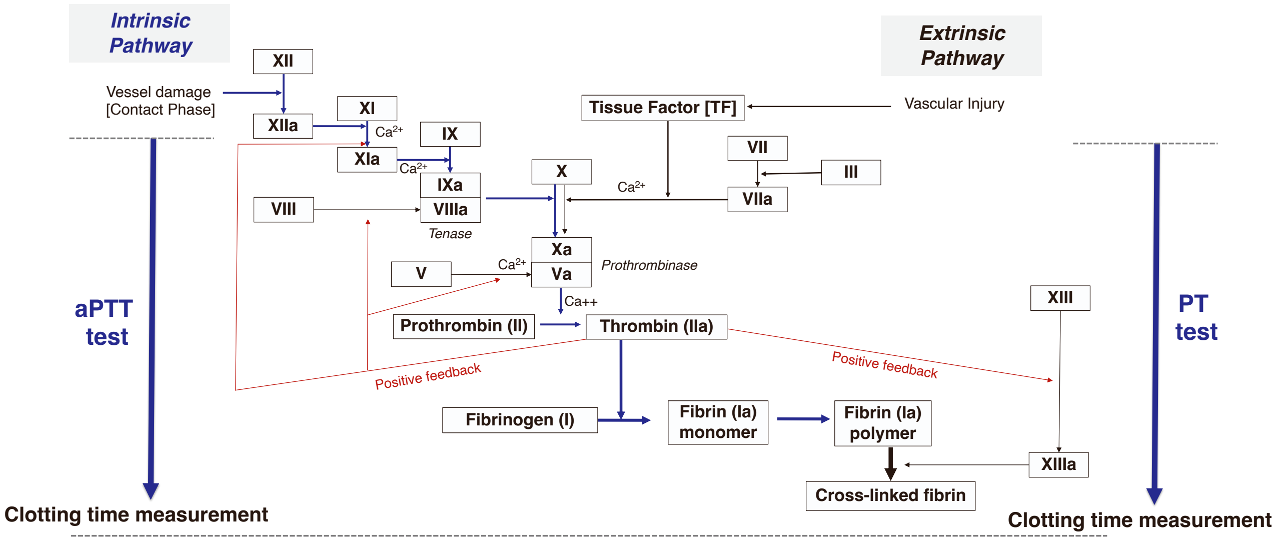

Figure S7. Blood coagulant factors in a cascade work as secondary hemostasis in a body. This can be separated into the intrinsic pathway and extrinsic pathway, which can be investigated by aPTT and PT test, respectively. In the intrinsic pathway, in brief, FVIII exhibits cofactor function (active FVIII; FVIIIa) when activated by a small amount of thrombin or active factor X (FXa) in the early phase of the coagulation reaction (coagulation initiation phase), and it dramatically promotes factor X (FX) activation catalyzed by a complex with active factor IX (FIXa) as tenase. The resulting FXa is complexed with its cofactor active factor V (FVa) as prothrombinase, converts prothrombin to thrombin and thrombin is explosively amplified by its positive feedback actions to upstream. This is called a thrombin burst. The large amount of thrombin produced rapidly converts fibrinogen into fibrin, the final product of the coagulation reaction. Note, aPTT reagent activates FXII and FIX, and PT reagent activates FVII to occur fibrin aggregation. From the reaction with FX to downstream is a common pathway of intrinsic and extrinsic pathways.

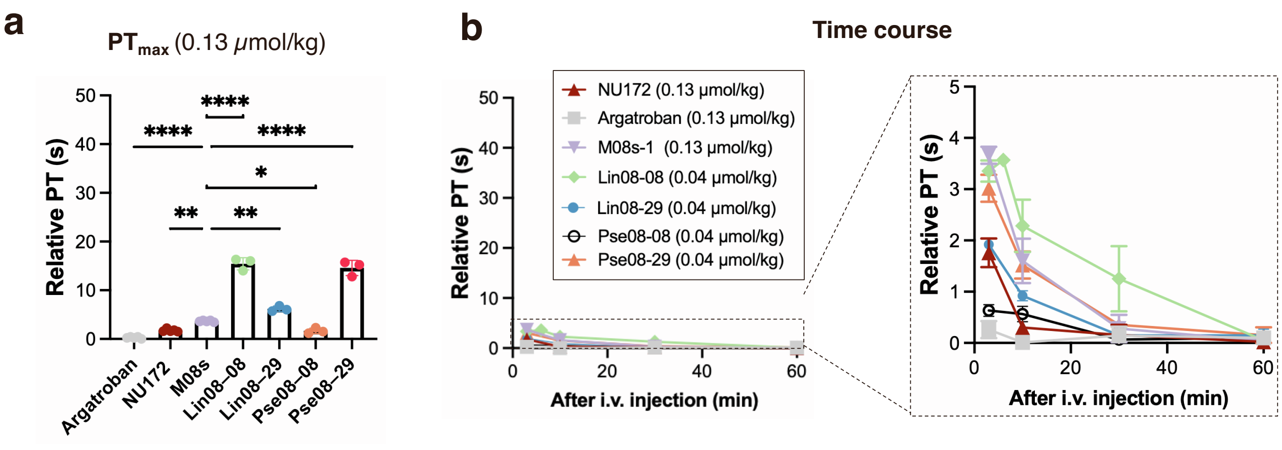

Figure S8. *Ex vivo* anticoagulant effect analyzed by prothrombin (PT) assay. (a) The max-anticoagulant effect at 3 min timepoint is shown. The dose of injected samples was aligned to 0.13 μmol/kg for all compounds. The statistical significance was tested using the t-test; (*); $p < 0.1$, (**); $p < 0.01$, (****); $p < 0.0001$. (b) Time course of clotting time plotted against collected serum after the injection at several timepoints. Relative PT was gained by subtracting the treated data by 11.7 sec, an averaged value of aptamer (–) as control PT in a triplicate.

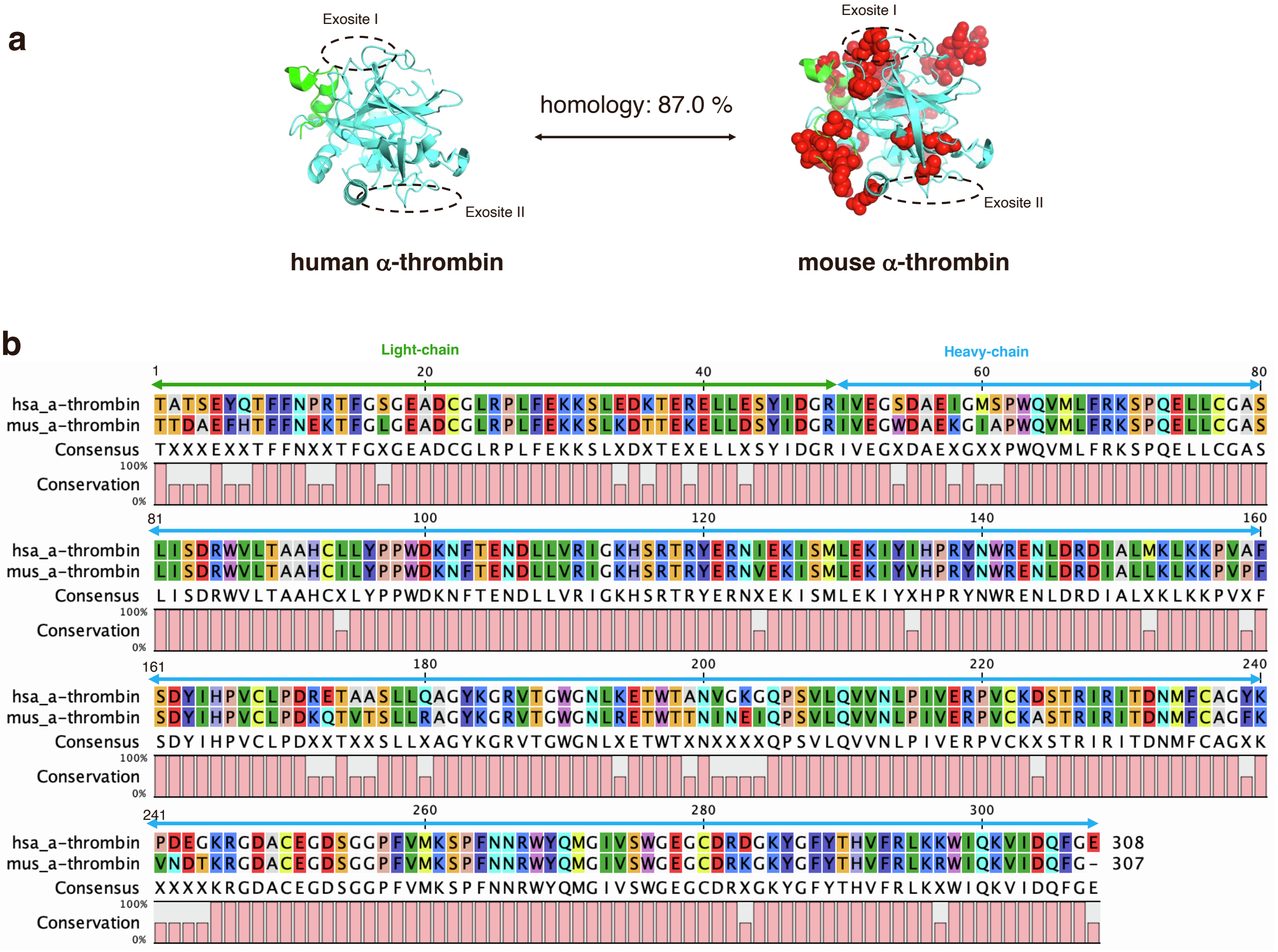

Figure S9. (a) Homology assessment of human (pdb: 3u69) and mouse a-thrombin (pdb: 2pux). The homology was determined by pairwise alignment of EMBL-EBI site. Red sphere in mouse thrombin represent mutated amino acids. (b) Alignment of human and mouse a-thrombin sequence. The amino acid sequences were gained from UNIPROT as prothrombin sequences, then alignment was performed by CLC sequence viewer ver. 7. has, homosepience; mus, mouse. The mutations at 40 sites were found by the comparison.

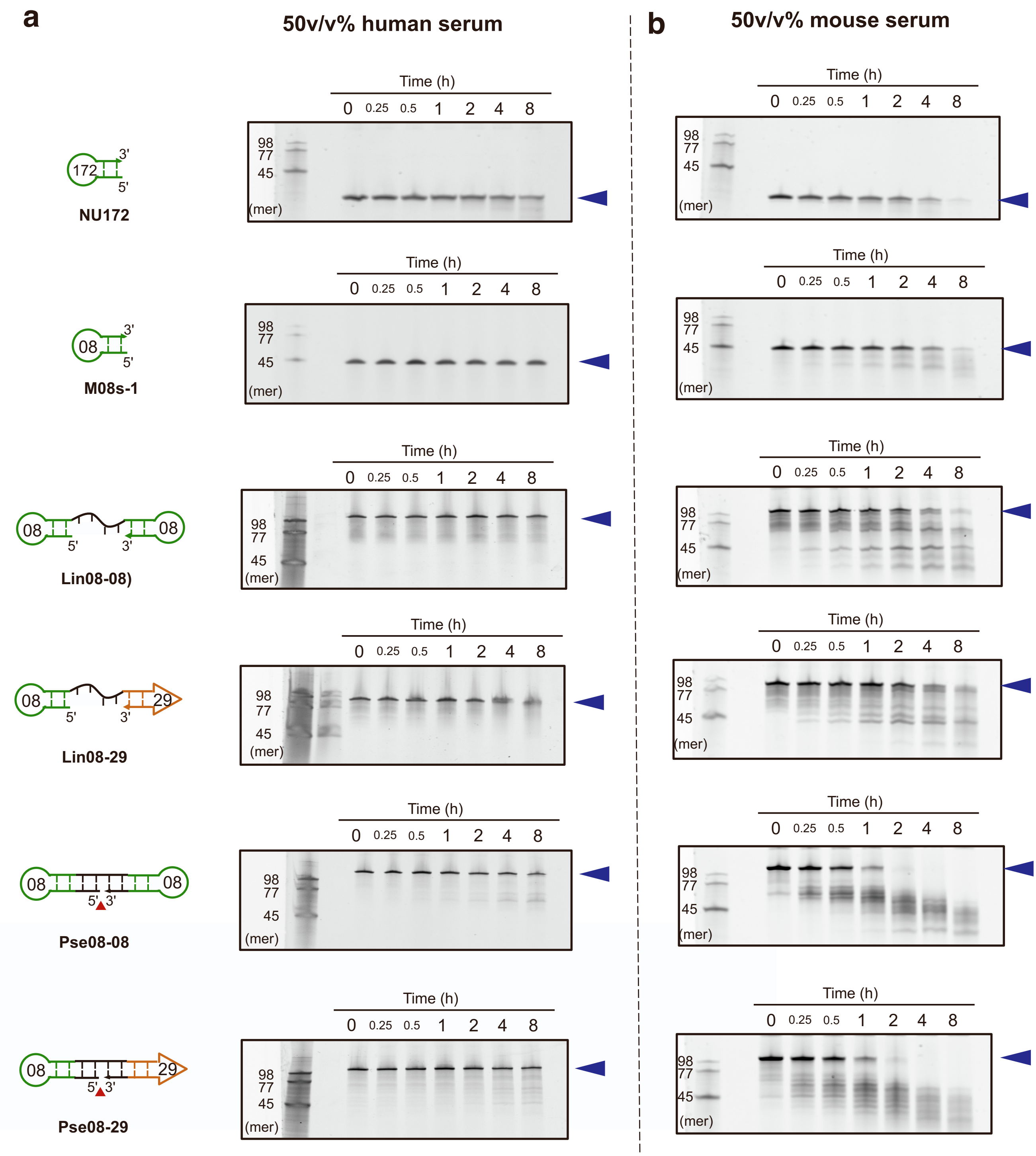

Figure S10. Stability of anti-thrombin DNA aptamers in human serum and mouse serum. (a) Results in 50v/v% human serum. (b) Results in 50v/v% mouse serum. The initial concentration of the aptamers was 2 μM.

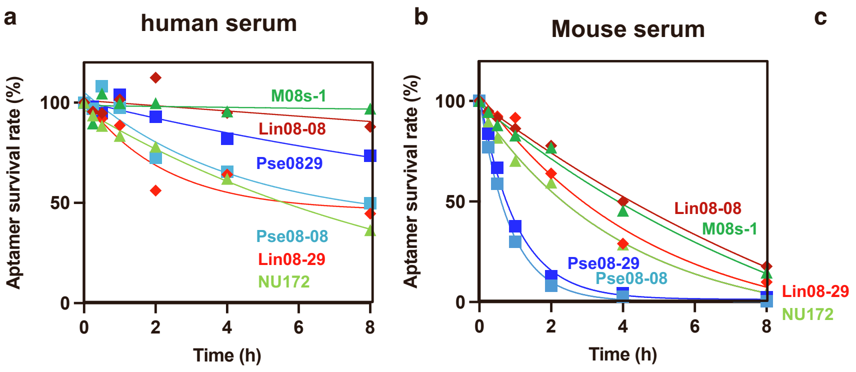

c

Aptamer	Half life (**h**)	
	human serum	mouse serum
NU172	6.9	2.4
M08s-1	>8.0	3.8
Lin08-08	>8.0	4.0
Lin08-29	7.8	3.3
Pse08-08	>8.0	0.6
Pse08-29	>8.0	0.7

Figure S11. Summary of serum stability test of the aptamers. (a) Stability in 50v/v% human serum. (b) Stability in 50v/v% mouse serum. (c) Half-lives of the aptamers.

a

Entry		Sequence	Length (mer)
	**Pse(08-29)**	5'-GTCACTAGAGGTCAGATGATGGGGATGGGGGGTTGGAGGAATGGATGACCT–(17mer linker)–TBA29-CGTAAATCA-3'	106
1	**[M08s full]c**	3'-TCCAGTCTACTACCCCTACCCCCCAACCTCCTTACCTACTGGA-5'	43
2	**[M08s G4]c**	3'-TCCAGTCTACTACCCCTACCCCCCAACC-5'	28
3	**[M08s G2]c**	3'-TCCAGTCTACTACCCCTACCCC-5'	22
4	**[M08s G0]c**	3'-TCCAGTCTACTACC-5'	14
5	**[M08s critical]c**	3'-CCTACCCCCCAACC-5'	14
6	**[Lin-M08s G0]c**	3'-CAGTGATCTCCAGTCTACTACC-5'	22
7	**[Lin-M08s G2]c**	3'-CAGTGATCTCCAGTCTACTACCCCTACCCC -5'	30
8	**[Lin-M08s G4]c**	3'-CAGTGATCTCCAGTCTACTACCCCTACCCCCCAACC-5'	36
9	**[Lin-M08s full]c**	3'-CAGTGATCTCCAGTCTACTACCCCTACCCCCCAACCTCCTTACCTACTGGA-5'	36

b

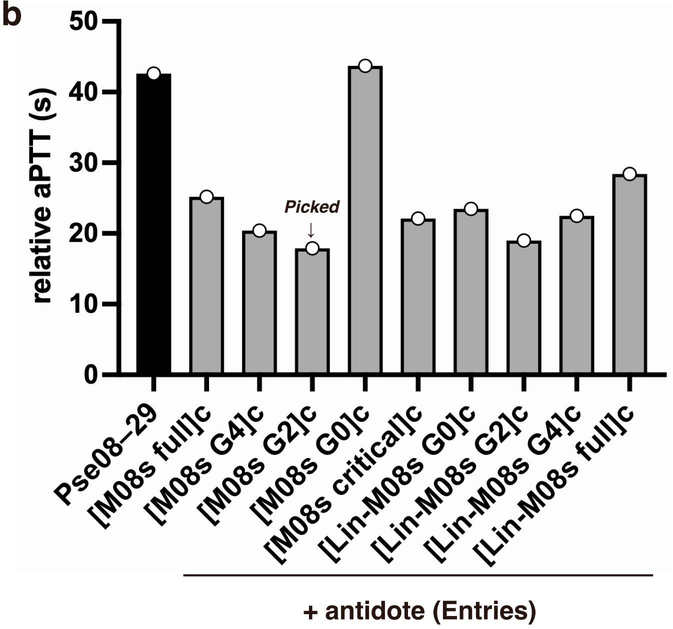

Figure S12. Screening of antidotes against Pese08-29 using aPTT test. (a) Sequence list of designed antidotes. Green, M08s-1 sequence; red, G-quadruplex forming deoxyguanines predicted by QGRS mapper. (b) Evaluation of antidotes against Pse08-29 using aPTT test in human plasma. Aptamer concentration, 0.33 μM; antidote concentration, 5.33 μM (16-fold to aptamer); temperature, 37 ˚C in the assay. Relative clotting time in human plasma was gain by subtraction of the treated data by 28.7sec as the value of aptamer (–).

Table S1. Summary of oligonucleotide sequence in this study.

Note	Aptamer	Length (mer)	ssDNA Sequence (5' to 3')
Monomer	TBA29	29	AGTCCGTGGTAGGGCAGGTTGGGGTGACT
	TBA15	15	GGTTGGTGTGGTTGG
	NU172	26	CGCCTAGGTTGGGTAGGGTGGTGGCG
	RE-31	31	GTGACGTAGGTTGGTGTGGTTGGGGCGTCAC
	M08s–scm	43	AGGTCAGATGTGGTGGTGGAGTAGGTGAGAGAAGAGGGTGCCT
	M08s	43	AGGTCAGATGATGGGGATGGGGGGTTGGAGGAATGGATGACCT
Duplex-linker optimization	Pse08-29 0 bp	72	AGGTCAGATGATGGGGATGGGGGGTTGGAGGAATGGATGACCTAGTCCGTGGTAGGGCAGGTTGGGGTGACT
	TA-5 bp	82	TAAGGTCAGATGATGGGGATGGGGGGTTGGAGGAATGGATGACCTTATATAGTCCGTGGTAGGGCAGGTTGGGGTGACTATA
	TA-9bp	90	TATAAGGTCAGATGATGGGGATGGGGGGTTGGAGGAATGGATGACCTTATATATATAGTCCGTGGTAGGGCAGGTTGGGGTGACTATATA
	TA-13bp	98	TATATAAGGTCAGATGATGGGGATGGGGGGTTGGAGGAATGGATGACCTTATATATATATATAGTCCGTGGTAGGGCAGGTTGGGGTGACTATATATA
	TA-17bp	106	TATATATAAGGTCAGATGATGGGGATGGGGGGTTGGAGGAATGGATGACCTTATATATATATATATATAGTCCGTGGTAGGGCAGGTTGGGGTGACTATATATATA
	TA-21bp	114	TATATATATAAGGTCAGATGATGGGGATGGGGGGTTGGAGGAATGGATGACCTTATATATATATATATATATATAGTCCGTGGTAGGGCAGGTTGGGGTGACTATATATATATA
	AT/GC-5bp	82	AAAGGTCAGATGATGGGGATGGGGGGTTGGAGGAATGGATGACCTTTACGAGTCCGTGGTAGGGCAGGTTGGGGTGACTCGT
	AT/GC-9bp	90	ATCAAGGTCAGATGATGGGGATGGGGGGTTGGAGGAATGGATGACCTTGATTTACGAGTCCGTGGTAGGGCAGGTTGGGGTGACTCGTAA
	AT/GC-13 bp	98	CAGTCAAGGTCAGATGATGGGGATGGGGGGTTGGAGGAATGGATGACCTTGACTGATTTACGAGTCCGTGGTAGGGCAGGTTGGGGTGACTCGTAAAT
	AT/GC-17bp = Pse08-29	106	GTCACTAGAGGTCAGATGATGGGGATGGGGGGTTGGAGGAATGGATGACCTCTAGTGACTGATTTACGAGTCCGTGGTAGGGCAGGTTGGGGTGACTCGTAAATCA
	AT/GC-21bp	114	CACTAGACTGAGGTCAGATGATGGGGATGGGGGGTTGGAGGAATGGATGACCTCAGTCTAGTGACTGATTTACGAGTCCGTGGTAGGGCAGGTTGGGGTGACTCGTAAATCAGT
Reported dimeric aptamers	TBA15-A15-TBA29 (TBA15-TBA29 heterodimer)	59	GGTTGGTGTGGTTGGAAAAAAAAAAAAAAAAGTCCGTGGTAGGGCAGGTTGGGGTGACT
	RA36 (TBA15 homodimer)	31	GGTTGGTGTGGTTGGTGGTTGGTGTGGTTGG
	0/0A$_2$/0A$_4$ strand-A	51	AAGTCCGTGGTAGGGCAGGTTGGGGTGACTTAGATGAGGCACGTCCCGCTC
	0/0A$_2$/0A$_4$ strand-B	47	CGAGTATAGGAATGGTGCGTAGGTTGGTGTGGTTGGGGCGCACAAAA
	0/0A$_2$/0A$_4$ strand-C	41	AACATTCCTATACTCGGAGCGGGACGTGAACTACCTCATCT
**Dimeric aptamers in this study**	Lin08-08	103	AGGTCAGATGATGGGGATGGGGGGTTGGAGGAATGGATGACCTTTTTTTTTTTTTTTTTTAGGTCAGATGATGGGGATGGGGGGTTGGAGGAATGGATGACCT
	Lin08-29	89	AGGTCAGATGATGGGGATGGGGGGTTGGAGGAATGGATGACCTTTTTTTTTTTTTTTTTTAGTCCGTGGTAGGGCAGGTTGGGGTGACT
	Pse08-08	120	GTCACTAGAGGTCAGATGATGGGGATGGGGGGTTGGAGGAATGGATGACCTCTAGTGACTGATTTACGAGGTCAGATGATGGGGATGGGGGGTTGGAGGAATGGATGACCTCGTAAATCA
	Pse08-29 (= AT/GC-17bp)	106	GTCACTAGAGGTCAGATGATGGGGATGGGGGGTTGGAGGAATGGATGACCTCTAGTGACTGATTTACGAGTCCGTGGTAGGGCAGGTTGGGGTGACTCGTAAATCA
**Antidotes in this study**	[M08s-1 critical]c	14	CCAACCCCCCATCC
	[M08s-1 full]c	43	AGGTCATCCATTCCTCCAACCCCCCATCCCCATCATCTGACCT
	[M08s-1 G0]c	14	CCATCATCTGACCT
	[M08s-1 G2]c	22	CCCCATCCCCATCATCTGACCT
	[M08s-1 G4]c	28	CCAACCCCCCATCCCCATCATCTGACCT
	[Lin-M08s-1 G0]c	22	CCATCATCTGACCTCTAGTGAC
	[Lin-M08s-1 G2]c	30	CCCCATCCCCATCATCTGACCTCTAGTGAC
	[Lin-M08s-1 G4]c	36	CCAACCCCCCATCCCCATCATCTGACCTCTAGTGAC

Supplemental References

1. Hu, X. *et al.* Structure-Guided Designing Pre-Organization in Bivalent Aptamers. *J. Am. Chem. Soc.* **144**, 4507–4514 (2022).
